# A Game-Theory Method to Design Job Rotation Schedules to Prevent Musculoskeletal Disorders Based on Workers’ Preferences and Competencies

**DOI:** 10.3390/ijerph16234666

**Published:** 2019-11-22

**Authors:** Sabina Asensio-Cuesta, Juan M. García-Gómez, José-Luis Poza-Luján, J. Alberto Conejero

**Affiliations:** 1 Instituto de Tecnologías de la Información y Comunicaciones (ITACA), Universitat Politècnica de València, Camino de Vera s/n, 46022 València, Spain; juanmig@ibime.upv.es; 2 Instituto Universitario de Áutomática e Informática Industrial, Universitat Politècnica de València, Camino de Vera s/n, 46022 València, Spain; jopolu@disca.upv.es; 3 Instituto Universitario de Matemática Pura y Aplicada, Universitat Politècnica de València, Camino de Vera s/n, 46022 València, Spain; aconejero@upv.es

**Keywords:** job rotation, preferences, competencies, ergonomics, Gale-Shapley method, game-theory, musculoskeletal disorders

## Abstract

Job rotation is an organizational strategy based on the systematic exchange of workers between jobs in a planned manner according to specific criteria. This study presents the GS-Rot method, a method based on Game Theory, in order to design job rotation schedules by considering not only workers’ job preferences, but also the competencies required for different jobs. With this approach, we promote workers’ active participation in the design of the rotation plan. It also let us deal with restrictions in assigning workers to job positions according to their disabilities (temporal or permanent). The GS-Rot method has been implemented online and applied to a case in a work environment characterized by the presence of a high repetition of movements, which is a significant risk factor associated with work-related musculoskeletal disorders (WMSDs). A total of 17 workstations and 17 workers were involved in the rotation, four of them with physical/psychological limitations. Feasible job rotation schedules were obtained in a short time (average time 27.4 milliseconds). The results indicate that in the rotations driven by preference priorities, almost all the workers (94.11%) were assigned to one of their top five preferences. Likewise, 48.52% of job positions were assigned to workers in their top five of their competence lists. When jobs were assigned according to competence, 58.82% of workers got an assignment among their top five competence lists. Furthermore, 55.87% of the workers achieved jobs in their top five preferences. In both rotation scenarios, the workers varied performed jobs, and fatigue accumulation was balanced among them. The GS-Rot method achieved feasible and uniform solutions regarding the workers’ exposure to job repetitiveness.

## 1. Introduction

Although robots are now widely used in modern industry, human resources still represent an essential part of it. As the present industrial processes are characterized by a high degree of production flexibility, the workforce also needs to be flexible.

Job rotation is an organizational strategy based on the systematic exchange of workers between jobs in a planned manner. A design of job rotation is to plan and schedule the assignment and sequencing jobs between workers. An optimal program depends on the variability in the assignment and balancing workload and tasks among operations, which does not protect a particular employee but rather reduces the risk exposure of all workers [1].

Job rotation contributes to preventing work-related musculoskeletal disorders (WMSDs), increasing workers’ knowledge and competence, and also developing social relationships [2]. Workers tend to perceive job rotation as a helpful method of enhancing satisfaction, productivity, and product quality [3]. Job rotation has recently been proposed as a suitable intervention for managing the exposure to age-related risk factors [4]. A study of 422 assembly lines in the Hyundai Motor Company concluded that job rotation was especially effective in preventing WMSDs in workers less than 45 years of age, while its effects were not evident for those of 45 years or older [3].

Moreover, job rotation can facilitate both progressive returns of workers in the process of rehabilitation of WMSDs, as the integration of workers with other types of disabilities. The job rotation allows assigning workers to positions compatible with their limitations, controlling the time they are exposed to factors of risk that are especially harmful to them [5].

According to the European Foundation for the Improvement of Living and Working Conditions (2015) [6], 43% of workers in the EU practiced job rotation in 2015. The rotation is more frequent in the activities of Health (63%) and Construction (62%), and, by occupation, between the Service workers and sellers (54%) and the Operators of facilities and machinery (53%). Rotation may involve the performance of tasks with similar content, or others that require different skills or qualifications. Moreover, the rotation can be decided by the head or superior, or more autonomously by the workers themselves. In centers with ten or more employees, 15% of workers routinely rotate between tasks that require different skills and are decided with some autonomy by them. Another 21% of workers benefit from the rotation between varied tasks, although decided by the hierarchy of the work center. Job rotation schedules should also prevent workers from spending their whole time at workplaces with high demands and ensure a balanced distribution of risks among individual work assignments [6]. To maximize job rotation efficiency, the schedule should be designed according to multiple criteria [7], such as workload levels and task characteristics [8]. Different studies have addressed the problem of job rotation under diverse approaches: ergonomic, biomechanical, organizational, cognitive-mental, environmental, safety, and aging, either jointly or independently [9].

The job rotation scheduling problem is a combinatorial problem, whose cost increases exponentially with the number of workers. As off-the-shelf software is not usually able to solve instances of practice-relevant size, customized algorithms need to be designed [10].

Genetic algorithms have been used to find rotation agendas: Carnahan et al. in 2000 [11] used them to obtain job rotation scheduling with ergonomic objectives; Asensio-Cuesta described their application to design rotations that minimize cumulative worker fatigue [12]; considering ergonomic and competence criteria [7]; and to prevent work-related musculoskeletal disorders in repetitive work applying job rotation [13]. Other approaches in the literature include Otto and Scholl in 2013 [10], who applied a heuristic and taboo search algorithm for finding rotations that reduce ergonomic risks; Song et al. in 2015 [14] proposed a job rotation scheduling algorithm for minimizing accumulated workload per body parts; Yoon et al. in 2016 [8] developed a mathematical model to calculate rotation schedules to reduce cumulative workload from the successive use of the same body region; in Boenzi et al. in 2016 [15], the obtained schedules sought the best solutions in a sub-group of operators requiring lower risk exposure; and finally, Botti et al. in 2017 [4] proposed a mathematical model to design activity schedules for elderly workers exposed to repetitive work risks. Sana et al., in 2019 [16], recently proposed a mathematical model of the problem of job rotation considering ergonomic aspects in repetitive works, lifting tasks, and awkward postures in manufacturing environments with high variability.

Job rotation can also be seen as an organizational work intervention that can benefit from a participatory ergonomics (PE) approach. PE stands for actively involving workers in developing and implementing workplace changes to improve productivity and reduce risks to safety and health [17]. PE states that workers are best placed to identify and analyze problems and to develop and implement solutions that are effective in reducing injury risks, improving productivity, and, last but not least, being acceptable to those affected. PE also contributes to reducing the number of musculoskeletal injuries, creating a more human-centered work, improving the organizational climate, and helping health promotion within the work environment [18].

Game theory consists of a collection of models that helps to understand situations in which decision-makers interact [19]. Game theory is the mathematical study of strategy and conflict, in which an agent’s success in making choices depends on the choice of others [20]. They can be understood as a dynamical graph system [21].

The Gale-Shapley algorithm (GS) [22] is fundamental in game theory, gives a solution to the stable marriage problem, and is simple to apply. When preferences are strict, the deferred acceptance algorithm yields a unique stable matching in O(n^2^) time. Since it was first applied to solve the college admissions problem, it has been applied to a myriad of contexts, and it also gave birth to a new theory of market design [23]. A recent description of the formulation and solution to some of these problems can be found in [24,25,26]. The GS algorithm has been applied to analyzing the capacity of sharing in a network of enterprises [27], and also for decision-making of reconfigurable manufacturing systems [28]. However, to the best of our knowledge, so far, it has not been used to solve the job rotation problem.

In the job rotation problem, a company requires its employees to swap their jobs (possibly on a regular basis) in order to avoid, for instance, exposure to monotonous [29] or risky ergonomic tasks. For the first rotation, a set of N workers has to be matched to a set of N jobs. In the next rotation, new matchings between workers and jobs have to be found so that no worker repeats his previous job position. The quest for such matches can be understood as a stable marriage problem [22]. The elements of each set have an ordered list of preferences with respect to the members of the other set. A solution to this problem consists of finding pairs of elements (job-worker) with one element from each set that are stable marriages, i.e., the breakup of two pairs cannot yield a new pair with better preferences for both members.

This paper proposes the GS-Rot method based on the Galey-Shapley algorithm [22], which is intended to design job rotation schedules based on game theory. The GS-Rot method can design job rotation schedules from two points of view, either by giving priority to workers’ job preferences or to their level of competence to carry out the jobs. The proposed method promotes the workers’ active participation in the design of the rotation plan by considering their preferences for job assignments. A case study was carried out in an assembly line of repetitive jobs as an example of its application, the main conclusions of which were: its flexibility was able to generate job rotation schedules according to different scenarios (with or without rotation, and also giving priority to workers’ preferences for assignments or to their skills); its efficiency in terms of computational cost for finding feasible schedules; its capability to obtain schedules that balance exposure to repetitiveness; and last but not least, its flexibility to introduce new assignment criteria.

## 2. Materials and Methods

### 2.1. Problem Definition

The problem to be solved consists of scheduling R rotations in the assignment of N workers to N job positions. For its design, we will consider workers’ preferences for the jobs and their degree of competence for each job. We will also consider organizational restrictions and an evaluation of the ergonomic quality of the proposed schedules.

We also want the worker-job assignments to be stable, i.e., there must be no couple of worker-job pairs such that, once broken, we could make a new, more profitable worker-job pair from the point of view of worker preferences and level of competence for the job.

### 2.2. Adaptation of the Gale-Shapley Algorithm (GS) to the Job Rotation Problem

The GS algorithm is applied to two sets of equal size, X and Y, in which each element in X has an ordered list of preferences with respect to the members of Y, and conversely. The X and Y sets are not treated equally. In each iteration, the algorithm gives priority to the preferences of the members of one of the sets. The elements in this set will be called requesters, and the others will be called requested. Different admissible solutions to the problem will be found according to which set belongs to the requesters.

To be matched, the GS algorithm requires the preference lists of each requester and requested. In the adaptation of the GS algorithm to the job rotation problem, we first consider that the requesters will be the workers, and the requested will be the job positions. For the converse assignment, we proceed in a similar way.

In the GS-Rot method, each worker will rank all the job positions according to his preferences. This will be called the *list of preferences* of job positions for worker i. We denote the order k-th of preference of worker i for job j as P(i,j).

We will also consider an ordered list of preferences of workers for each job j, which will be based on the skills required to carry it out. This will be called the *list of competencies* for job position j. We denote the order k-th of competence for worker i at job j as C(i,j). We will also add a *table of ergonomic fitness* in which the ergonomic risk of each worker in each job position has been computed. The ergonomic fitness of worker i at job position j is denoted by EF(i,j) so that any given rotation can be scored in terms of ergonomic quality.

The GS algorithm only provides a single assignment of workers to job positions. However, for the job rotation problem, we require a list of paired assignments for each rotation. After each rotation, we will thus modify the lists of job positions and competencies. If only one assignment of workers to jobs were to be implemented, there would be no job rotations.

### 2.3. General Description of the GS-Rot Method

Let us assume that we have N workers and N job positions and we have to schedule R rotations. We consider the lists of preferences and competencies as given. Initially, we take them without any modification. As priority will be given to the workers’ preferences, it will be called an assignment based on preferences.

Let us introduce a counter for the rotations, named *r*, and initialize it at 1. The method works as follows:

Step 1. Let us compute the *r* rotation. We first assign each worker to the job position of his first preference.

Step 2. Job positions with two or more requests will be provisionally assigned to the most suitable worker, i.e., the one who ranks highest in the list of competencies for that job. The other workers are considered as rejected.

Step 3. For each rejected worker in the previous step, we assign him/her to the next job position in his/her list of preferences. Those workers whose request has not been rejected in the previous step keep their assignment.

Step 4. We repeat the combination of Steps 2 and 3 until no worker is rejected. In this last case, we have already obtained the *r* rotation of the agenda. We denote the job assigned in rotation *r* to worker *i* as *A(i,r).*

Step 5. Once we have assigned the workers to jobs for rotation *r*, we will then update the preferences and competencies lists of every worker before returning to Step 1 and starting to compute the next rotation *r* + 1, as long as *r* ≤ R. Penalties are introduced in both lists to avoid re-assigning a worker to the same job as before.

**Figure 1:**
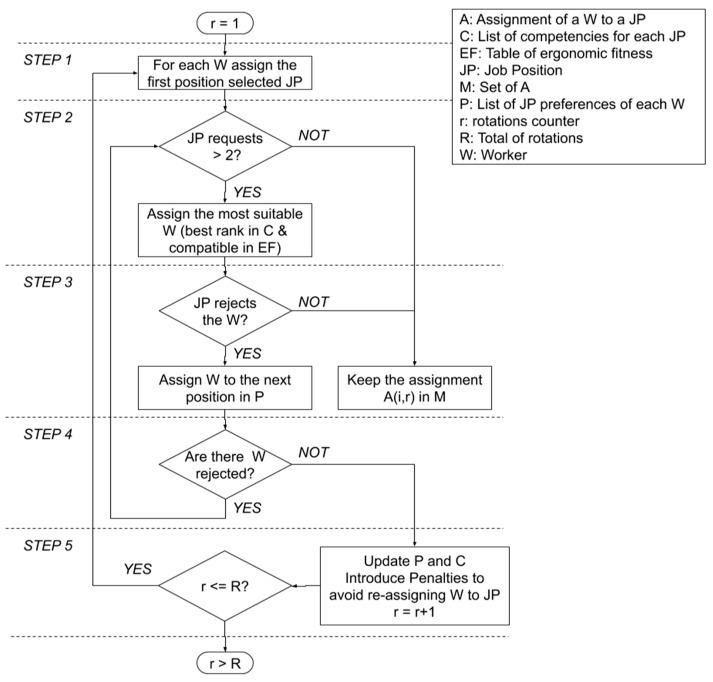
Flow chart of Gale-Shapley (GS)-Rot method.

Conversely, if the requesters are the job positions and the workers are the requested, we will call it an *assignment based on competencies*. The steps of the GS-Rot method are similar to the previous ones, but in this case, the job positions choose the workers. When a number of requests coincide, the workers’ preferences are consulted. This strategy gives preference to the competence level of the workers as regards their preferences. Figure 1 illustrates the flow chart of the GS-Rot method.

### 2.4. Definition of the Worker’s Preference Lists

We denote the order of preference of job j for worker i as P(i,j). We assign 1 to his first preference, 2 to the second, and so on. When N is low, we can rank “each worker’s” preference and competence for each job position. When N is high, and it is not feasible to individually rank all elements, we propose that workers evaluate each job position between 1 and T, where T indicates the number of subgroups of preferred jobs.

When a worker assigns the maximum score to a job, it may indicate that he has experience in this position and that he considers that he is qualified for the job. However, it may also indicate that he wants to do it for a change from his last job or to be assigned to a new job in which he can learn new competencies. Once a worker has been evaluated for all the positions, the positions are ranked from highest to lowest. In the case of tied evaluations, the order is randomized. For example, If the positions are P_0_, …, P_5_, and the grades for these positions are indicated between brackets P_0_(1), P_1_(2), P_2_(2), P_3_(3), P_4_(3), P_5_(3), then positions P_3_, P_4_, and P_5_ are tied, as well as P_0_ and P_2_. We break the ties by assigning random numbers to each one and ordering them according to this last number.

### 2.5. Definition of the Worker’s Competencies Lists

For the definition of the list of competencies, we propose to number (Ns) all the skills required for any job. For every skill s, we compute a score SkC(j,s), which determines if skill s is required for job j. For instance, we can score each skill for every job as 1, if necessary, and 0 if not necessary. Later, we score the workers’ ability for each skill required for each job; WSk(i,s) stands for worker i’s ability at skill s.

In the next example, we score as 1 if the worker has this skill and 0 if not.

Once we have graded the job skills and the workers level of skills for each job, we compute the competency level of every worker i for each job j, denoted by CL(i,j), as follows, see (Equation (1)):

(1) $$\mathrm{CL}\left(i,j\right)=\sum_{s=1}^{N_{s}}\mathrm{SkC}\left(j,s\right)\mathrm{WSk}\left(i,s\right).$$

Then we rank all workers from the highest to the lowest level of competence for every job position obtaining the competence list, denoted as C(i,j), in the example showed in Table 1 with j = 1, C(1,1) = 2, C(2,1) = 1, C(3,1) = 3. Table 1 gives an example of this computation. As previously indicated, we undo ties using random numbers.

### 2.6. Definition of the Ergonomic List of Jobs

In order to evaluate the fitness of a solution in terms of ergonomic quality, we needed to evaluate the ergonomic risk level of all job positions, and we denoted the score for the ergonomic risk of type e in job j as ER(j,e). This can be done attending to one or more risk factors, depending on the planner’s choice. For instance, for working environments with repetitiveness, the ergonomic list could include a score to indicate the presence of risks associated with each job. This can be obtained by applying Sue Rodger’s method [30] or the OCRA method [31]. If several factors are considered, we should homogenize the risk assessments to be comparable. Such values will be called the ergonomic list of job positions.

### 2.7. Penalties for the Assignments

Two types of penalties will be introduced in the GS-Rot method for assignments of workers to job positions:

(1) Penalties for taking a job position already taken: after obtaining an assignment in the r rotation, we update the competence lists introducing penalties for workers in job positions that they have already taken. This is done in order to force workers not to repeat the same positions in the next rotation: If a worker is assigned to job position j in rotation r, then we move him to the last position in the competence list of the job position j for the rotation *r* + 1. We also update the preference list of that worker in the same way by setting job positions already taken as the last preference of each worker.
(2) Penalties introduced by the planner: the planner can explicitly prohibit some assignments or indicate the maximum time that a job position can be taken by a worker. This is useful in order to include medical prohibitions or recommendations. If one assignment is forbidden, then we consider the next position in the worker’s preference list. If a worker does not find an acceptable assignment, this will lead to an undefined state in the solution given by the method, according to the degree of restriction of the penalties.

### 2.8. Fitness Evaluation of the Solution

The total fitness of a solution, E, will indicate the quality of the job assignments to workers in each rotation. This is done by simultaneously considering the fitness of preferences (E_p_), competencies (E_c_), and ergonomic criteria (E_e_). Equations (2)–(5) show a proposal of how fitness can be obtained. If we want to favor some criteria over others, some weights can be introduced in (2). As can be noted, the lower the value of the fitness, the better the solution.

(2) $$E=E_{p}+E_{c}+E_{e}$$

We recall that *A(i,r)* stands for the job position assigned in rotation r to worker i. The fitness of preferences and competencies will be given by

(3) $$E_{p}=\sum_{r=1}^{R}\sum_{i=1}^{N}P\left(i,A\left(i,r\right)\right),$$

(4) $$E_{c}=\sum_{r=0}^{R}\sum_{i=0}^{N}C\left(i,A\left(i,r\right)\right)$$

We denote the ergonomic fitness of a solution as E_e_. This weighs the risk to which the worker is exposed, plus the accumulated fatigue. For its computation, let us consider that we are going to use Ne different risk criteria. We also make D(r) stand for the duration of the rotation r without pauses. E_e_ is calculated for each worker (i) according to the exposure to each risk (e), the time exposed to it (D(r)), and the number of rotations performed previously (r). Thus, the worker exposure to risk increases with time exposed (D(r)), and it is higher with existing accumulative fatigue due to previous rotations (r).

By ER(i,A(i,r),e), we denote the level of ergonomic risk of type e to which worker i is exposed in rotation r when assigned to job A(i,r) according to the ergonomic list of job positions. For the computation of the ergonomic fitness, we consider that it is proportional to the exposure time and accumulative fatigue represented by introducing factors D(r) and r.

(5) $$E_{e}=\overset{R}{\underset{r=1}{\sum }}\overset{N}{\underset{i=1}{\sum }}\overset{N_{e}}{\underset{e=1}{\sum }}\mathrm{ER}\left(i,\;A\left(i,r\right),e\right)\times D\left(r\right)\times r+\overset{R}{\underset{r=2}{\sum }}\overset{N}{\underset{i=1}{\sum }}\overset{N_{e}}{\underset{e=1}{\sum }}F\left(i,A\left(i,r\right),\;r,e\right)$$

For the computation of E_e_ we have also taken into account the variability and the recovering of accumulated fatigue [13]. F(i,A(i,r),r,e) denotes the accumulated fatigue of worker i with respect to the risk e, when doing the job prescribed in rotation r + 1, after doing the job in rotation r, which depends on the variation of the risk level exposure when changing jobs. The ergonomic risk score of type e to which worker i is exposed when assigned to job A(i,r) in rotation r will be denoted by ER(i,A(i,r),r,e), which takes numerical values (scores) representing low (L), medium (M) or high (H) level of risk. We consider that there is no fatigue in the first rotation. Fatigue decreases when the worker is exposed to lower risk and increases in positions of medium or high risk. It also depends on the risks involved in the previous job, so that the lower the fatigue, the higher the ergonomic fitness. According to the previous and the next level of risk, we set 5 different fatigue levels: I(g), with g from 1 to 5. Its definition is given in Equation (6).

(6) $$F\left(i,r,e\right)=\{\begin{array}{c} I\left(1\right)\mathrm{if}\;\mathrm{ER}\left(i,A\left(i,r-1\right),\;r-1,e\right)=L\;\;\;\mathrm{or}\;\;\mathrm{ER}\left(i,A\left(i,r\right),r,e\right)=L\\ I\left(2\right)\mathrm{if}\;\mathrm{ER}\left(i,A\left(i,r-1\right),r-1,e\right)=M\;\mathrm{and}\;\mathrm{ER}\left(i,A\left(i,r\right),r,e\right)=M\\ I\left(3\right)\mathrm{if}\;\mathrm{ER}\left(i,A\left(i,r-1\right),r-1,e\right)=H\;\mathrm{and}\;\mathrm{ER}\left(i,A\left(i,r\right),r,e\right)=M\\ I\left(4\right)\mathrm{if}\;\mathrm{ER}\left(i,A\left(i,r-1\right),r-1,e\right)=M\;\mathrm{and}\;\mathrm{ER}\left(i,A\left(i,r\right),r,e\right)=H\\ I\left(5\right)\mathrm{if}\;\mathrm{ER}\left(i,A\left(i,r-1\right),r-1,e\right)=H\;\mathrm{and}\;\mathrm{ER}\left(i,A\left(i,r\right),r,e\right)=H \end{array}$$

In order to clarify the results, we will call the second term of (5) the accumulated fatigue of the rotation (denoted by F_a_).

### 2.9. Case Study

The GS-Rot method was used to obtain a rotation plan for *N* = 17 workers and jobs in a work environment characterized by the presence of a high repetition of movements, which is a major risk factor associated with work-related musculoskeletal disorders (WMSDs), taking into account a job’s repetitiveness risk level, the workers’ preferences and competencies, as well as some organizational restrictions.

We also wanted to evaluate the quality of the rotation agendas in terms of exposure to repetitiveness with a rotation every 2 h in an 8-h day, so that R = 4. We considered the following increments for the computation of the fatigue level in (Equation (6)): I(1) = 0, I(2) = I(3) = 2, I(4) = 3, and I(5) = 4 [13].

The workers ranked the job positions between 1 (lowest) and 5 (highest) according to their preferences. To obtain each worker’s preference list, we ordered the jobs from the highest to the lowest, randomly undoing ties. The results are indicated in Table 2.

We also identified a set of skills required for the jobs considered in the rotation: standing, exerting force standing still, exerting force in movement, driving vehicles, working at heights, reasoning/taking complex decisions, responsibility, initiative/autonomy, seeing from a distance, hearing, locating the direction of sound, writing, using a keyboard, using a mouse, computing capacity. These skills are scored with 1 if they are required for the job and 0 if not. Each worker was also evaluated for these skills in the same way, with 1 if he had it, and 0 if not. Combining both scores, as in Equation (1), we obtain the competence level of every worker for every job and the competence lists, as was done with worker preferences. The competence lists are showed in Table 3.

We also set 14 penalties: workers 6 and 10 cannot drive and cannot be assigned to job 5. Worker 13 cannot take job 6 since he cannot work at heights. The planner also decided that worker 3 cannot do jobs 1, 5, 6, 7, 8, 9, 10, 12, 13, 16, and 17 more than 4 h per day, but can combine them, for example, he can do job 1 for 2 h and job 5 for 2 h. In order to evaluate the workers’ cumulative repetitiveness of the solutions, we used Sue Rodgers’ method [30] to obtain a severity level for every job in terms of strength, duration, and frequency required for every part of the body in carrying out each task. According to the score obtained, the jobs were classified into 3 severity levels: Low (2), Medium (5), and High (7). Jobs 13 and 17 were identified as high, jobs 1, 7, 8, 10, 11, 12 as medium, and jobs 2, 3, 4, 5, 6, 9, 14, 15, and 16 as low. Other methods, such as OCRA, could have been applied in that context to assess the repetitiveness risk level [31].

## 3. Results

In this case study case, the GS-Rot method was implemented in JavaScript online software. To evaluate the method’s capacity for generating rotations, we compared the results of four different scenarios:

(1) Assignment by considering workers’ preferences as requesters without rotations (AP)
(2) Assignment by considering job competencies as requesters without rotations (AC)
(3) Assignment by considering workers’ preferences as requesters with rotations (APR)
(4) Assignment by considering job competencies as requesters with rotations (ACR)

In all the scenarios, GS-Rot was able to obtain acceptable assignments (Table 5) in a short time (mean 10.5 ms) on a 3.4 GHz Processor with 3.5 GB of RAM memory.

### 3.1. Fitness Analysis

The solution with the best fitness score was AP (E = 1317), while ACR and APR had the worst fitness score (E = 1418 and 1429, respectively). The best score for the fitness of preferences was obtained by the AP (E_p_ = 84) and the worst by AC (E_p_ = 398). The best fitness with competencies was obtained by AC (E_c_ = 134) and the worst by APR (E_c_ = 427). The best ergonomic fitness was obtained for the agendas obtained through ACR (E_e_ = 808) and APR (E_e_ = 810), since the part of the score due to fatigue in these scenarios was lower than the scenarios without rotation, (F_a_ = 64) for the ACR and (F_a_ = 64) for the APR case. All of this data is shown in Table 4 and Table 5.

When the method gave priority to workers’ preference with respect to the competence level, the results were in agreement with it. These solutions presented a worse score for E_c_. The situation is symmetrical with the converse case.

When introducing rotations in the scenarios driven by workers’ preferences or competencies, as expected, the variability of the jobs done by each worker is higher, so that, the cumulative fatigue is lower than in the non-rotational cases.

### 3.2. Analysis of Preferences

In the AP scenario, all the workers (100%) were assigned a job in their top five preferences, and 41.18% obtained their first option. Of the jobs, 47.04% were assigned to workers at the top of their competence list, and 76.45% in the top 10 places. When introducing rotations in APR, almost all the workers (94.11%) got an assignment among their top five preferences, with 35.29% given their first option. Likewise, 48.52% of job positions were assigned to workers in the top five of their competence lists, with 79.39% in the top 10.

**Figure 2:**
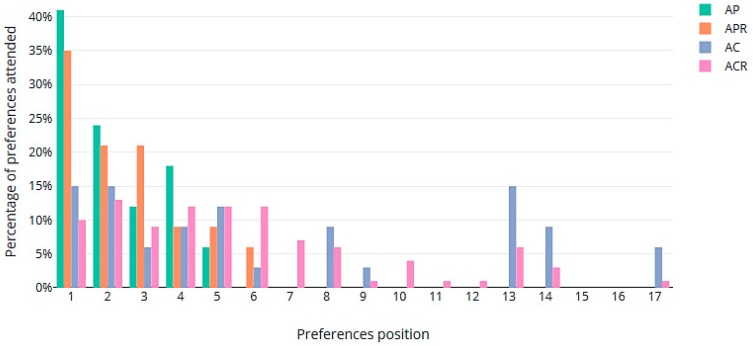
Percentages of preferences satisfied in the 4 scenarios.

As expected, the worst results in terms of preferences were obtained when the assignments prioritized competencies (scenarios AC and ACR). In AC, although 55.88% of the workers got one of their top five preferences, some were assigned to lower-ranked places: 2.94% of the workers obtained their sixth preference. Other positions with the corresponding percentage of workers were: positions 8 (8.82%), 9 (2.94%), 13 (14.71%), 14 (8.82%), and 17 (5.88%). The rate of assignments to the top five preferences (55.87%) was maintained in ACR when the rotations were included. Here, we also have other assignments in positions: 6 to 14 and 17. All of this data is summarized in Figure 2.

**Figure 3:**
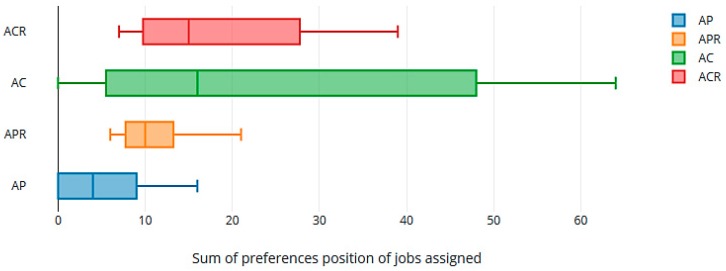
Box graph of the sum of preference position of jobs assigned.

**Figure 4:**
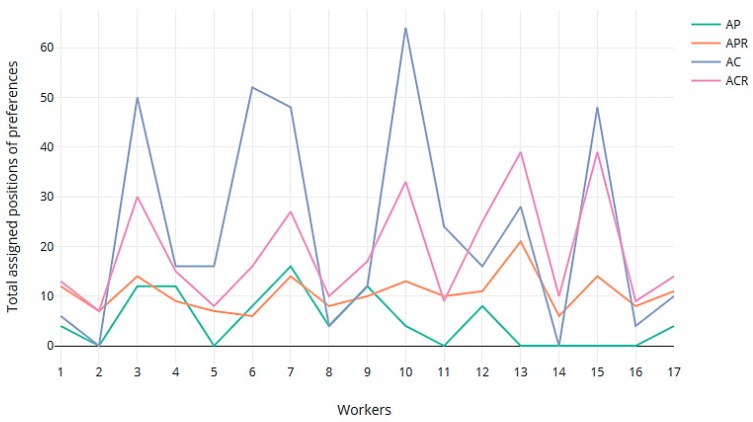
For each worker, the sum of positions in their list of preferences of the assigned jobs, when the assignment prioritized the worker’s preferences without rotations (AP) and with rotations (APR); and when the assignment prioritized the worker’s competencies without rotations (AC) and with rotations (ACR).

We also computed the sum of the positions in their respective preference lists of the jobs that were assigned to all the workers in all the rotations (Figure 3). As can be seen, the GS-Rot method achieved more uniformity in the degree of satisfying preferences when the rotation gave priority to the preferences. Figure 4 gives the total sum of preferences in the 4 rotations for every worker. High values in total assigned positions of preferences (peaks in the graph of Figure 4) indicate the presence of less preferred assignments by workers in the assignment. The GS-Rot method provided more uniformity in the level of satisfied preferences in AP and APR than in AC and ACR.

### 3.3. Analysis of Competencies

In the AC case, most of the workers were in their top five of their competence lists (85.29%), with 32.35% assigned to the first option. The rest of the workers were assigned to an option in the 6th (5.88%), 7th (5.88%), or 8th (2.94%) place. However, when prioritizing competencies, in AC, only 58.88% of the workers got assignments in their top five preferences (in contrast to the 100% in AP), and 78.58% got an assignment in their top 10 preferences.

**Figure 5:**
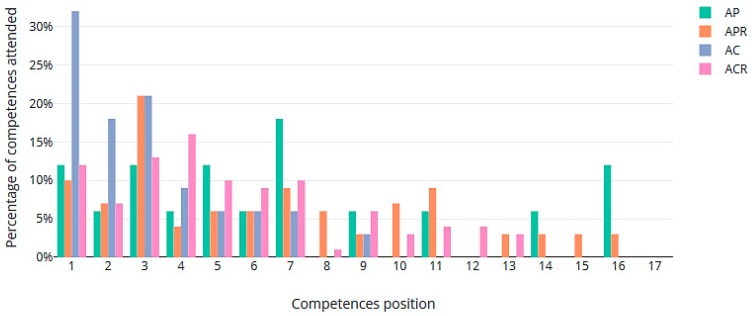
Percentages of competencies satisfied in the 4 scenarios.

When introducing rotations in ACR, 58.82% of workers got an assignment among their top five competence lists, and 11.76% achieved their first option. No workers were assigned to the last four places in their competence list (Figure 5). With rotation under competence priority (ACR), the satisfied workers’ preferences were lower than with APR, as expected. In ACR, 55.87% of the workers achieved jobs in their top five places, the rest were assigned jobs between preferences 6 and 17 (only 1.47% were assigned to their last preference), and 86.74% were in their top 10.

**Figure 6:**
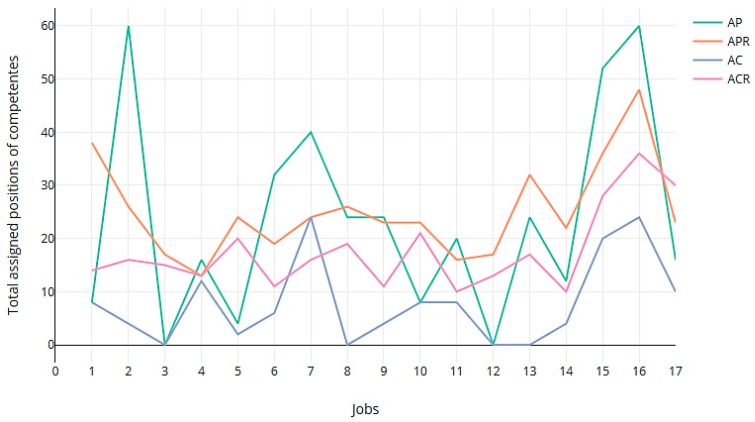
For each job, the sum of positions in their list of competencies of the assigned workers, when the assignment prioritized workers’ competencies without rotations (AC) and with rotations (ACR), and when the assignment prioritized workers’ preferences without rotations (AP) and with rotations (APR).

In general, the sums are lower for the AC and ACR than for AP and APR (Figure 6). It can be seen again that the GS-Rot method achieves uniformity in the level of satisfied competencies in AC and ACR. The peaks in Figure 6 show the number of workers assigned to the last place in their competence list.

**Figure 7:**
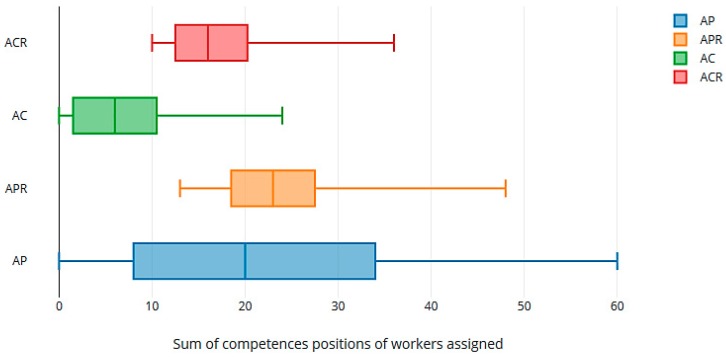
Box graph of the sum of assigned places in workers’ competencies lists.

For every job, we also computed the sum of the places in the workers’ competence lists assigned to them in all the rotations. These results are shown in Figure 7. As we can see, the GS-Rot method achieves more uniformity in the level of satisfied competencies in AC and ACR than in AP and APR.

### 3.4. Ergonomic Analysis

We finally computed the accumulated fatigue per worker (AFW) after each rotation scenario as the sum of the Sue Rodgers level of risk of every position multiplied by the length of the rotation and the position in the rotation (Equation (5)).

**Figure 8:**
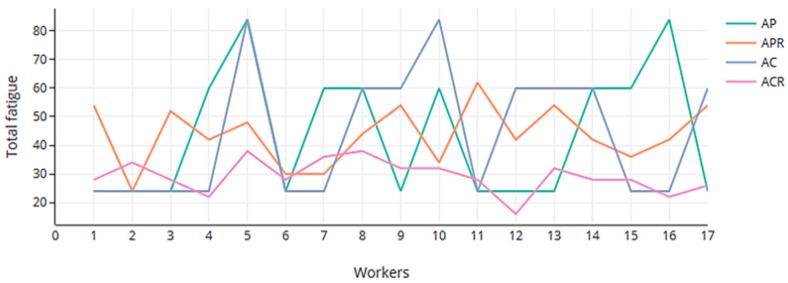
Accumulated fatigue by workers in each scenario.

**Figure 9:**
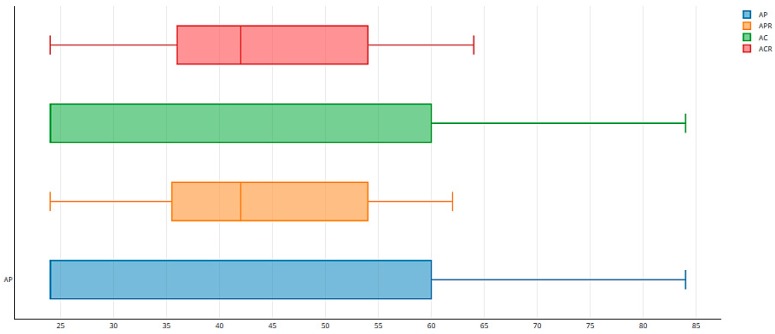
Box graphic of accumulated fatigue by workers.

Figure 8 shows the results of the risk of repetitiveness accumulated by each worker for different scenarios. It can be seen that the scenarios with rotations (APR and ACR) are able to spread the risk among the workers. However, in AP and AC, without rotation, some workers would appear to be over-fatigued (workers 5 and 16 in AP, workers 5 and 10 in AC). The distribution of the accumulated fatigue is shown in Figure 9 and also illustrates how rotation is able to balance the fatigue within workers.

## 4. Discussion

The GS-Rot method has been proved to be a useful tool for designing job rotation schedules based on worker’s preferences, required competencies, and ergonomics. The method provides job rotation schedules in acceptable short computing time so that planners are able to recalculate solutions to deal with any changes in production or human resources that affect the number and type of workers and jobs involved in the rotation.

GS-Rot can also find different acceptable solutions for a group of workers and job positions, according to whether the priority is given to job preferences or to maximizing their level of competence, with or without rotations. In the present case study of 17 workers and jobs, feasible job rotation schedules were obtained in a short time. In the rotation scenarios, with priority for both preferences and competencies, the method can introduce variability of the jobs done by each worker, while fatigue accumulation is lower than in the non-rotational scenarios. The workers did not consecutively repeat assignments, and the rotation schedules spread out repetitiveness among the workers. In this case, the GS-Rot method achieved uniformity in the level of satisfying preferences. However, uniformity decreased when the rotation gave priority to competence over job preferences. The rotations (APR and ACR) balanced the workers’ fatigue accumulation much better than the scenarios with no rotation (AP and AC).

It should be remembered that the workers’ participation in the rotation plan by considering their preferences can be of paramount importance for facilitating their acceptance of the solution and its deployment. It is worth mentioning that GS-Rot is not limited to generating agendas based on preferences or competencies. It can also introduce new assignment criteria in the requesters and requested lists. Other criteria can also be considered for planning the assignments, such as experience in the job, former injuries, training, and medical recommendations, or combinations of the foregoing.

With the job, rotation makes it possible to assign workers to workstations compatible with their limited capacities and to restrict the time that they are exposed to risk factors to which they are especially sensible [5]. The GS-Rot method allows obtaining solutions for disabled workers’ integration by applying penalties to certain assignations to avoid a worker to be assigned to a job that cannot perform due to physical, psychological, or communication limitations. However, it does not imply that positions considered “light” are assigned to a single worker, with the consequent rejection of other workers.

Finally, we should also mention that the system for introducing penalties is still open and subject to the inclusion of new criteria for computing the updated lists. For instance, they could be updated after each rotation according to the accumulated ergonomic risk, which would reduce the risk of injuries due to ergonomic factors. In future studies, we intend to apply the GS-Rot method to environments with multiple ergonomic risk factors.

## 5. Conclusions

In this study, we have described the new method GS-Rot to calculate job rotation by considering not only workers’ job preferences but also the competencies required for different jobs. Moreover, the method can also deal with restrictions in assigning workers to job positions, that could be useful for disabled (permanent or temporary) workers integration. The workers themselves can actively participate in designing the rotation plan taking into consideration their job assignations preferences. Mover, we have assessed the feasibility of the method by applying it in a case study in a work environment characterized by the presence of a high repetition of movements. Job rotation schedules are calculated in a short time, and almost all the workers (94.11%) were assigned to one of their top five preferences. When jobs were assigned according to competence, 58.82% of workers got an assignment among their top five competence lists. The GS-Rot method achieved uniform solutions as regards the workers’ exposure to job repetitiveness and cumulative fatigue.

Finally, we are aware that the study is limited with regard to the jobs and workers involved. Although the results are promising, more cases of study will be needed to validate the method. Likewise, to validate the post-implementation result of the rotation schedules proposed by the GS-Rot method remains pending, and this will require the collection of data after its application. These data should be compared with the musculoskeletal incidents before the schedule adopted. Due to the cumulative nature of the WMSDs, a sufficiently long period of observation will be necessary (at least two years) [5]. Furthermore, the degree of satisfaction of workers should be compared to previous solutions in which their preferences were not considered. Additionally, we plan to improve the GS-Rot online tool developing a user-centered interface to be usable and accepted by planners in real scenarios.

## Figures and Tables

**Table 1:** Example of the computation of workers’ competence for a specific job position j. In this case, we have three workers, i = 1, 2, 3, and also 3 competencies, s = 1, 2, 3. Here, worker 2 has the highest competency level, followed by workers 1 and 3.

Competencies	SkC(j,s)	WSk(1,s)	WSk(2,s)	WSk(3,s)
Driving forklift truck (s = 1)	1	1	1	0
Computing capacity (s = 2)	0	1	0	1
Previous experience (s = 3)	1	0	1	0
Competence level (CL)		1 × 1 + 0 × 1 + 1 × 0 = 1	1 × 1 + 0 × 0 + 1 × 1 = 2	1 × 0 + 0 × 1 + 1 × 0 = 0
Order by competence level		2º	1º	3º

**Table 2:** Workers’ preference lists (P). A value in row i, column j, P(i,j) indicates the position of preference for worker i of job j, e.g., in the first row worker 1 has job 4 as first preference (P(1,4) = 1) and job 5 as the second (P(1,5) = 2).

Jobs																		
**Workers**		**1**	**2**	**3**	**4**	**5**	**6**	**7**	**8**	**9**	**10**	**11**	**12**	**13**	**14**	**15**	**16**	**17**
	**1**	7	4	12	1	2	3	13	15	10	11	9	8	6	5	17	16	14
	**2**	12	7	1	3	4	6	8	13	14	15	16	17	10	2	5	9	11
	**3**	1	16	9	10	12	11	14	15	13	17	3	5	6	2	4	7	8
	**4**	9	5	12	11	10	8	7	6	4	3	2	1	14	13	16	15	17
	**5**	16	7	4	8	9	5	2	3	17	14	15	12	13	11	10	6	1
	**6**	17	8	4	1	10	11	9	6	7	5	3	2	13	12	16	14	15
	**7**	14	12	6	5	7	8	1	2	4	3	11	9	10	15	13	17	16
	**8**	2	3	5	9	8	12	14	13	17	7	10	11	1	6	4	15	16
	**9**	8	10	1	7	15	16	13	2	11	17	12	4	14	5	6	9	3
	**10**	6	8	15	7	12	11	14	2	9	16	1	5	17	3	13	10	4
	**11**	15	2	11	14	12	6	7	5	9	17	4	3	16	1	13	10	8
	**12**	1	2	8	13	12	4	10	11	6	7	5	16	17	14	15	3	9
	**13**	3	1	9	11	12	7	10	8	5	4	2	17	13	15	16	6	14
	**14**	3	5	10	11	6	9	7	13	16	1	2	17	12	14	15	4	8
	**15**	12	2	11	5	7	4	1	10	16	6	3	14	8	13	15	9	17
	**16**	12	11	7	2	6	4	5	10	13	9	3	16	1	14	17	8	15
	**17**	12	15	10	1	17	2	3	14	4	6	7	11	13	9	16	8	5

**Table 3:** Jobs competencies lists (C). A value in row i, column j, C(i,j) indicates the order of competence of worker i in job j, e.g., worker 1 is the 5th in the competence level for job 1 (C(1,1) = 5); and worker 1 is the 1st in the competence level for jobs 6 and 7 (C(1,6) = 1; C(1,7) = 1).

Jobs																		
**Workers**		**1**	**2**	**3**	**4**	**5**	**6**	**7**	**8**	**9**	**10**	**11**	**12**	**13**	**14**	**15**	**16**	**17**
	**1**	5	13	15	13	2	1	1	2	8	6	15	8	9	5	13	17	14
	**2**	10	1	1	1	10	12	4	3	10	8	2	14	11	3	12	4	4
	**3**	16	4	8	6	13	5	5	5	1	15	11	15	3	13	14	10	7
	**4**	4	2	2	2	3	7	12	9	7	7	14	5	4	10	1	2	3
	**5**	1	10	9	9	1	2	6	8	15	12	12	16	5	7	5	8	5
	**6**	9	7	6	7	14	10	16	12	16	11	6	4	15	8	15	7	6
	**7**	2	5	5	5	4	15	13	14	13	14	7	10	12	9	6	5	8
	**8**	3	6	4	3	8	3	7	13	2	2	5	12	17	1	11	9	10
	**9**	6	11	10	11	7	6	10	17	11	9	9	1	2	16	3	6	9
	**10**	13	9	11	10	17	16	17	7	17	16	17	9	1	14	16	11	11
	**11**	7	3	3	8	5	4	15	10	12	1	4	11	13	4	17	1	2
	**12**	11	17	17	16	9	13	8	11	4	5	3	2	14	6	4	16	15
	**13**	17	16	12	14	12	17	2	1	14	17	16	6	10	11	2	14	17
	**14**	12	12	14	17	15	14	3	6	5	3	1	17	6	12	7	12	12
	**15**	14	14	16	15	16	11	11	4	6	13	13	7	16	2	8	13	16
	**16**	8	8	7	4	6	8	14	15	9	10	10	3	7	15	9	3	1
	**17**	15	15	13	12	11	9	9	16	3	4	8	13	8	17	10	15	13

**Table 4:** Fitness values and execution time for rotation agendas obtained after applying the GS-Rot in the 4 scenarios.

Characteristic	Jobs AP	Jobs APR	Jobs AC	Jobs ACR
Fitness (E)	1317	1418	1369	1429
Preferences Fitness (Ep)	84	181	398	321
Competencies Fitness (Ec)	400	427	134	300
Ergonomic Fitness (Ee)	833	810	837	808
Fatigue (Fa)	89	66	93	64
Mean Execution Time (10 repetitions)	32.2 milliseconds	27.6 milliseconds	25.3 milliseconds	24.8 milliseconds

**Table 5:** Rotation agendas obtained after applying the GS-Rot in the 4 scenarios.

Workers	Jobs AP				Jobs APR				Jobs AC				Jobs ACR			
	R1	R2	R3	R4	R1	R2	R3	R4	R1	R2	R3	R4	R1	R2	R3	R4
**1**	5	5	5	5	5	6	14	13	5	5	6	6	5	6	1	14
**2**	3	3	3	3	3	14	4	15	3	3	3	3	3	14	4	15
**3**	15	15	15	15	15	13	16	1	9	9	7	7	9	13	16	17
**4**	9	9	9	9	9	10	12	2	2	2	2	2	2	1	12	9
**5**	17	17	17	17	17	7	6	8	17	17	5	5	17	3	6	7
**6**	11	11	11	11	11	12	3	4	16	16	16	16	16	12	11	4
**7**	4	4	4	4	4	8	9	5	15	15	15	15	15	4	3	5
**8**	1	1	1	1	1	15	13	3	1	1	1	1	1	15	2	3
**9**	12	12	12	12	12	3	15	17	12	12	12	12	12	17	15	1
**10**	8	8	8	8	8	17	2	14	13	13	13	13	13	16	8	2
**11**	14	14	14	14	14	2	17	12	6	6	17	17	6	2	14	11
**12**	16	16	16	16	16	1	11	9	11	11	11	11	11	10	9	8
**13**	2	2	2	2	2	16	8	7	8	8	8	8	8	7	5	13
**14**	10	10	10	10	10	11	1	16	10	10	10	10	10	11	7	16
**15**	7	7	7	7	7	5	10	6	14	14	14	14	14	8	10	12
**16**	13	13	13	13	13	4	5	11	4	4	4	4	4	5	13	6
**17**	6	6	6	6	6	9	7	10	7	7	9	9	7	9	17	10
